# Sexual Dimorphism in Swim Bladder Texture, Composition, and Muscle Nutrient Profile of Commercial-Sized Black-Spotted Croaker (*Protonibea diacanthus*)

**DOI:** 10.3390/ani16162534

**Published:** 2026-08-14

**Authors:** Cheng Peng, Jiahui Chen, Huayi Xue, Ningwen Zhang, Sen Li, Yaorong Wang, Yong Zhang, Shijia Hu

**Affiliations:** 1 Guangdong Key Laboratory of Animal Conservation and Resource Utilization, Guangdong Public Laboratory of Wild Animal Conservation and Utilization, Institute of Zoology, Guangdong Academy of Sciences, Guangzhou 510260, China; pengcheng@giz.gd.cn (C.P.); huayisher@sina.com (H.X.); rrrealname@163.com (N.Z.); 2 State Key Laboratory of Biocontrol, Guangdong Provincial Key Laboratory for Aquatic Economic Animals, Southern Marine Science and Engineering Guangdong Laboratory (Zhuhai), School of Life Sciences, Sun Yat-Sen University, Guangzhou 510275, China; chenjh563@mail2.sysu.edu.cn (J.C.); wangyr95@mail2.sysu.edu.cn (Y.W.); 3 Guangdong Havwii Agricultural Group Co., Ltd., Zhanjiang 524266, China; lisen@havwii.com.cn

**Keywords:** *Protonibea diacanthus*, sexual dimorphism, swim bladder, texture profile analysis, nutrient composition

## Abstract

The swim bladder (fish maw) of the blackspotted croaker (*Protonibea diacanthus*) commands a high price for males, yet the biological basis for this preference has remained unclear. We evaluated sexual dimorphism in commercially sized fish (*n* = 30 per sex) by comparing body weight, and assessed swim bladder texture and nutrient composition of both swim bladder and dorsal muscle in a subset of four fish per sex. Male swim bladders possess significantly superior textural properties than those of females, particularly in the key parameters of hardness (1804.33 ± 581.05 vs. 728.53 ± 46.81 gf), springiness (0.91 ± 0.03 vs. 0.73 ± 0.09), and chewiness (1161.18 ± 171.86 vs. 374.68 ± 97.72 gf) (all *p* < 0.05), whereas body weight, swim bladder weight, and total collagen content did not differ. Swim bladder amino acid and fatty acid profiles were largely similar between sexes, except for higher glutamic acid and leucine in males. In contrast, dorsal muscle proximate and nutrient profiles showed no significant differences between sexes, indicating nutritionally equivalent fish meat. These results demonstrate that sex critically affects swim bladder but not muscle quality, highlighting the need for further investigation into the mechanisms underlying these textural differences of swim bladder and explore potential dietary or genetic interventions to modulate swim bladder quality in aquaculture.

## Simple Summary

The black-spotted croaker is a large marine fish highly valued for its swim bladder. This gas-filled organ, dried and sold as “fish maw”, is a luxury food in Asian markets. Male fish maw often sells at much higher prices than female fish maw, but the reasons for this preference have remained unclear. To investigate, we compared male and female fish of commercial harvest size, measuring body weight, swim bladder weight, texture, and nutritional quality of both the swim bladder and the muscle fillet. We discovered that male swim bladders were significantly firmer, more elastic, chewier, and bounced back better after compression compared to female swim bladders, even though swim bladder weight and total collagen (the main structural protein) content were similar between the sexes. In contrast, the muscle meat from males and females was nutritionally identical. Our findings reveal that sex critically influences the texture of the swim bladder but not the quality of the meat, providing a scientific explanation for the market preference for male fish maw. This knowledge can help the fish maw industry grade products by sex and may encourage selective rearing strategies or feeding practices to enhance swim bladder quality in aquaculture.

## 1. Introduction

The black-spotted croaker, *Protonibea diacanthus* (family Sciaenidae), is a commercially important marine fish species widely distributed across the tropical Indo-Pacific region [1]. This large, fast-growing species can reach over 150 cm in total length and up to 45 kg in weight, with females maturing at two years of age at a total length of approximately 98 cm [1,2,3]. In addition to its rapid growth and large body size, *P. diacanthus* exhibits strong mobility, and tolerance to environmental variability, making it a promising candidate for offshore marine ranching [4,5,6]. As a high-value food fish, *P. diacanthus*, like other large sciaenid fishes, is well suited for value-added processing into products such as steaks, fillets, and meatballs [7]. However, its market value is driven primarily by its swim bladder, which is highly prized as a collagen-based functional food in Asian markets [8]. The dried swim bladder (fish maw) from *P. diacanthus* commands exceptionally high prices due to its desirable texture and nutritional properties, reaching 409.5 USD/kg [9]. Notably, in several sciaenid fishes, including *P. diacanthus*, the swim bladder of males is generally considered superior in quality and commands higher market prices than that of females, reflecting pronounced sexual differences in texture and structural properties [2,10,11]. Given the high commercial value of both its muscle and swim bladder, understanding whether these economically important traits differ between males and females is essential for optimizing aquaculture practices and product utilization.

Sexual dimorphism, the systematic phenotypic differences between males and females of a species, is a common phenomenon in teleost fishes [12]. These differences include body size, growth rate, coloration and various physiological and biochemical characteristics [13,14,15]. Among these traits, body size and growth rate represent the most prominent and economically relevant forms of sexual dimorphism in many commercially farmed fish species. A case of extreme sexual size dimorphism in farmed teleosts is the half-smooth tongue sole (*Cynoglossus semilaevis*), whose females grow two to four times faster than males and exhibit body size that are more than twice of males when harvesting [16,17]. Within the Sciaenidae family, sexual size and growth dimorphism has also been documented in several species. For example, in the yellow drum (*Nibea albiflora*), females grow 30% faster than males, and can reach market size in 18 months instead of 24 months in males [18,19]. Similarly, the large yellow croaker (*Larimichthys crocea*) exhibits considerable sexual size dimorphism under both cultured and natural conditions, with females growing faster than males [20]. Beyond body size and growth rate, sexual dimorphism in teleosts also extends to muscle nutrient composition. For instance, in golden pompano (*Trachinotus blochii*), the total amino acid, essential amino acid, and polyunsaturated fatty acid contents in the muscle of females are higher than those in males, suggesting that female fish generally possess higher nutritional value [21]. Such pronounced sexual dimorphism has made monosex culture economically advantageous, driving the development of sex control technologies and monosex breeding programs [19,22].

Recent studies have demonstrated significant sexual dimorphism in swim bladder texture properties in sciaenid species such as Chu’s croaker (*Nibea coibor*), where males exhibit higher hardness, springiness, chewiness, and shear force compared to females [10,11]. Proteomic analyses further suggest that these differences are closely associated with collagen fiber organization and composition, particularly the expression of structural proteins such as collagen XII [11]. Despite the growing number of research on sexual dimorphism in sciaenid fishes, comprehensive studies that evaluate body weight, swim bladder traits, size and texture of *P. diacanthus* remain lacking. Such integrative analyses are essential for understanding the full extent of sex-related differences and their implications for aquaculture production and product utilization. Therefore, the present study aims to systematically evaluate sexual dimorphism in commercial-sized *P. diacanthus* by comparing males and females with respect to body weight, swim bladder size and textural properties, and nutrient composition of swim bladder and muscle. The findings will provide valuable insights for sex-based resource utilization, selective breeding strategies, and value-added processing in the aquaculture industry.

## 2. Materials and Methods

### 2.1. Rearing Conditions and Sampling

Commercial-sized *P. diacanthus* were obtained from a commercial marine cage farm of Guangdong Havwii Agricultural Group Co., Ltd. (Zhanjiang, China). Fish were reared in sea cages under natural photoperiod and fed chopped trash fish daily at 3% of body weight. Water temperature ranged from 24.5 to 28.5 °C, salinity from 28 to 32‰, and dissolved oxygen was maintained above 5.0 mg/L throughout the rearing period. A random subset of 60 healthy fish (30 females and 30 males), all from the same batch and reared together in a single cage under identical environmental and feeding conditions, was harvested for subsequent analyses. Fish were euthanized by immersion in tricaine methanesulfonate (MS-222, 100 mg/L; Sigma-Aldrich, St. Louis, MO, USA). Each fish was weighed (body weight, BW, kg) and measured for body length (BL, cm). The abdominal cavity was opened, and the gonads (ovary or testis) were visually inspected to determine sex. The swim bladder was carefully dissected from each fish, cleaned of adhering adipose tissue and blood vessels, and dried with filter paper. Swim bladder weight (SW, g) was recorded, and relative swim bladder weight (RSW, %) was calculated as (SW/BW) × 100. For subsequent texture and nutrient composition analyses, the four females and four males whose body weight was closest to the median body weight of their respective sex were selected, and their swim bladders and dorsal muscle were sampled. Animal handling and experimental procedures were approved by the Animal Research and Ethics Committees of the Institute of Zoology, Guangdong Academy of Sciences (Approval No. GIZ20251014; Date: 14 October 2025).

### 2.2. Texture Profile Analysis (TPA) of Swim Bladder

For texture analysis, the swim bladder was cut into uniform rectangular specimens (approximately 5 cm × 5 cm) from the central region, avoiding the edges. Specimen thickness was measured with a digital caliper. TPA was performed using a Universal TA texture analyzer (Tengba, Shanghai, China) equipped with a cylindrical probe (36 mm diameter). Test parameters were set as follows: pre-test speed 0.3 mm/s, test speed 0.3 mm/s, post-test speed 0.3 mm/s, compression ratio 40% of original thickness, trigger force 5 gf (gram-force), and time interval 3 s between two compressions. The following texture parameters were derived from the force-time curve according to standard definitions [23]: hardness (gf; peak force of the first compression), springiness (ratio; recovery of sample height), cohesiveness (ratio; ratio of areas under the second and first compressions), chewiness (gf; hardness × cohesiveness × springiness), resilience (ratio; upstroke energy/downstroke energy of the first compression), and gumminess (gf; hardness × cohesiveness). Three replicate measurements were performed on each swim bladder (*n* = 4 per sex) at different positions with an interval time of 30 s, and the average value was used for statistical analysis.

### 2.3. Nutrient Composition Analysis

Nutrient composition was determined separately for dorsal muscle (white muscle from the epaxial region) and swim bladder (central portion). A portion of each sample was taken fresh for moisture content analysis. The remaining material was then freeze-dried and stored at −80 °C for all subsequent analyses. All proximate analyses were performed with four replicates per sex. All analytical procedures were conducted in accordance with the corresponding National Food Safety Standards of China (GB standards). Briefly, moisture was determined by oven-drying at 105 °C to constant weight (GB 5009.3) [24]. Ash was measured by incineration at 550 °C for 6 h (GB 5009.4) [25]. Crude protein was analyzed by the Kjeldahl method with a nitrogen-to-protein conversion factor of 6.25 (GB 5009.5) [26]. Crude lipid was extracted with petroleum ether using a Soxhlet system (GB 5009.6) [27]. Amino acid composition was determined after hydrolysis with 6 M HCl at 110 °C for 24 h under nitrogen by ion-exchange chromatography with post-column ninhydrin derivatization (GB 5009.124) [28]. Tryptophan was not determined due to degradation during acid hydrolysis. Protein quality indices, including EAA/TAA (%) and EAA/NEAA (%), were calculated. Total amino acids (TAA) were the sum of all quantified amino acids. Essential amino acids (EAA) comprised Thr, Val, Met, Ile, Leu, Phe, Lys, His and Arg (Trp not determined), and NEAA (non-essential amino acid) was derived as TAA minus EAA. Fatty acid composition was analyzed as fatty acid methyl esters (FAMEs, prepared using 14% boron trifluoride in methanol) by gas chromatography with flame ionization detection (GB 5009.168) [29]. In brief, FAMEs were separated on an SP-2560 capillary column (100 m × 0.25 mm i.d., 0.20 µm film thickness; Supelco, Bellefonte, PA, USA) with nitrogen as the carrier gas at a constant flow rate of 1.0 mL/min. The injector and detector temperatures were set at 270 °C and 280 °C, respectively. Peaks were identified by comparison with a standard FAME mixture (Supelco 37 Component FAME Mix, Supelco). Collagen content was determined by colorimetric measurement of hydroxyproline after acid hydrolysis (GB/T 9695.23) [30] and calculated as hydroxyproline content × 8.0, assuming 12.5% hydroxyproline in collagen [31,32,33], though collagen values thus derived should be viewed as estimates given potential species-specific variation in hydroxyproline content. Mineral element concentrations (Mg, Ca, Mn, Fe, Cu, Zn, Se, Cd, Pb) in swim bladder samples were determined by inductively coupled plasma mass spectrometry after microwave digestion (GB 5009.268) [34]. Mercury (Hg) was analyzed using a direct mercury analyzer (GB 5009.17) [35]. Results were expressed as mg/kg dry weight.

### 2.4. Statistical Analysis

All statistical analyses were performed using SPSS version 26.0 (IBM, Armonk, NY, USA) or R software (version 4.2.1). Data were expressed as mean ± standard deviation (SD). Comparisons between male and female groups were conducted using Welch’s *t*-test. Pearson or Spearman correlation analysis was performed to evaluate relationships between body weight and swim bladder size parameters. Statistical significance was set at *p* < 0.05. To aid interpretation of non-significant comparisons, standardized effect sizes (Cohen’s d) were considered alongside *p*-values.

## 3. Results

### 3.1. Body Weight and Swim Bladder Size

**Figure 1:**
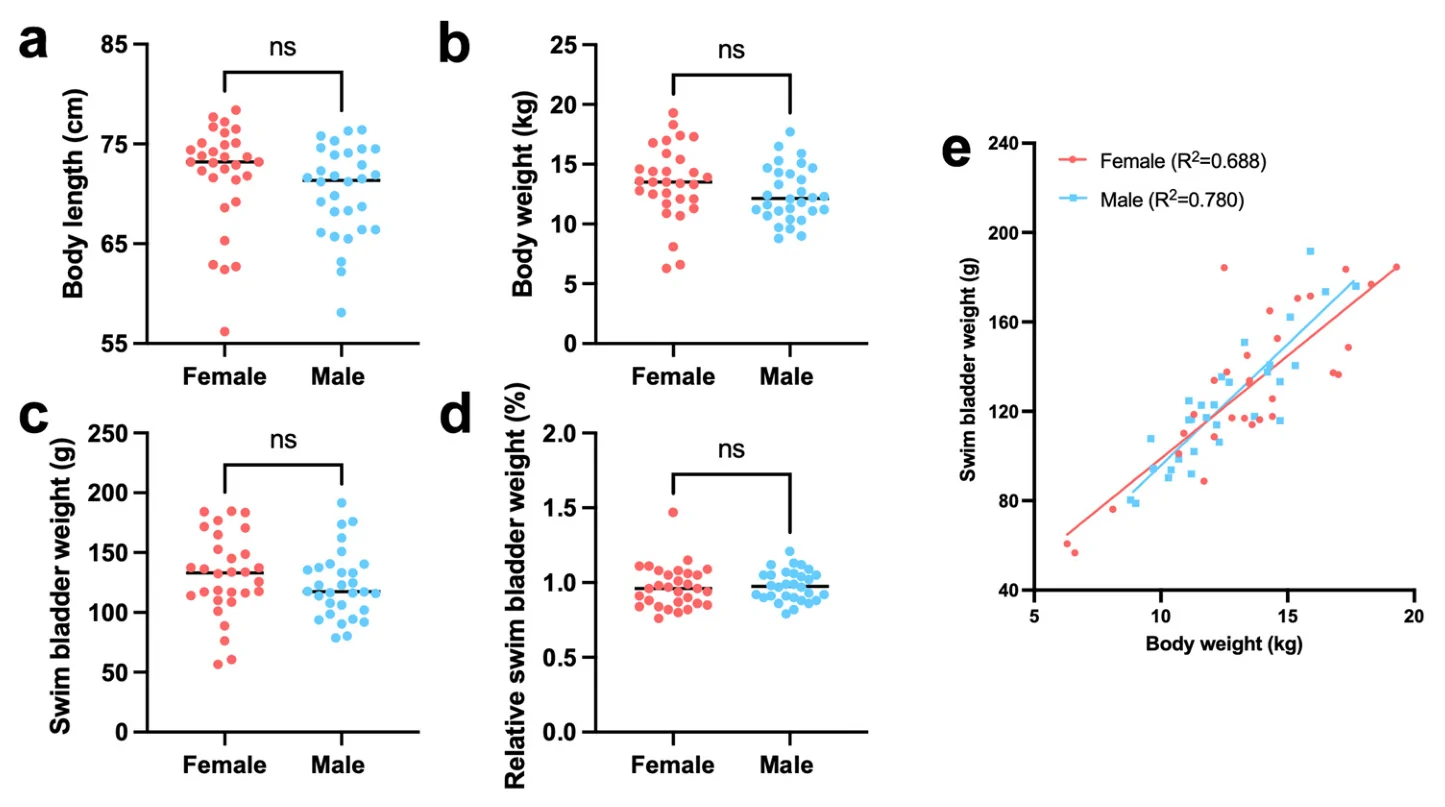
Morphometric comparison and SW-BW relationship in female and male Black-spotted Croaker (*Protonibea diacanthus*). (**a**) Body length (BL), (**b**) body weight (BW), (**c**) swim bladder weight (SW), and (**d**) relative swim bladder weight (RSW). Data are presented as mean ± SD with individual data points overlaid (*n* = 30 per sex). Statistical significance was assessed by independent samples *t*-test; “ns” denotes no significant difference (all *p* > 0.05). (**e**) Correlation between SW and BW in females (red) and males (blue). The coefficients of determination were R^2^ = 0.688 (females) and R^2^ = 0.780 (males), both significant at *p* < 0.001.

A total of 30 female and 30 male commercial-sized *P. diacanthus* harvested from a single cage were examined (Figure 1, Table S1). Female body weight (BW) ranged from 6.3 to 19.3 kg (mean ± SD: 13.5 ± 3.1 kg), while male BW ranged from 8.8 to 17.7 kg (12.5 ± 2.3 kg; Figure 1b). Although females were, on average, heavier than males, the difference did not reach statistical significance (*p* = 0.17). Total length followed a similar pattern (female: 71.8 ± 5.3 cm vs. male: 70.1 ± 4.6 cm; *p* = 0.20; Figure 1a). Female swim bladders were slightly heavier than those of males (130.7 ± 34.4 g vs. 122.9 ± 28.1 g; Figure 1c), though this difference was also non-significant. When adjusted for body weight, relative swim bladder weight (RSW) was comparable between sexes (female: 0.97 ± 0.14% vs. male: 0.98 ± 0.10%; Figure 1d). Correlation analysis revealed that swim bladder weight was positively correlated with body weight in both sexes (Figure 1e).

### 3.2. Texture Properties of the Swim Bladder

**Figure 2:**
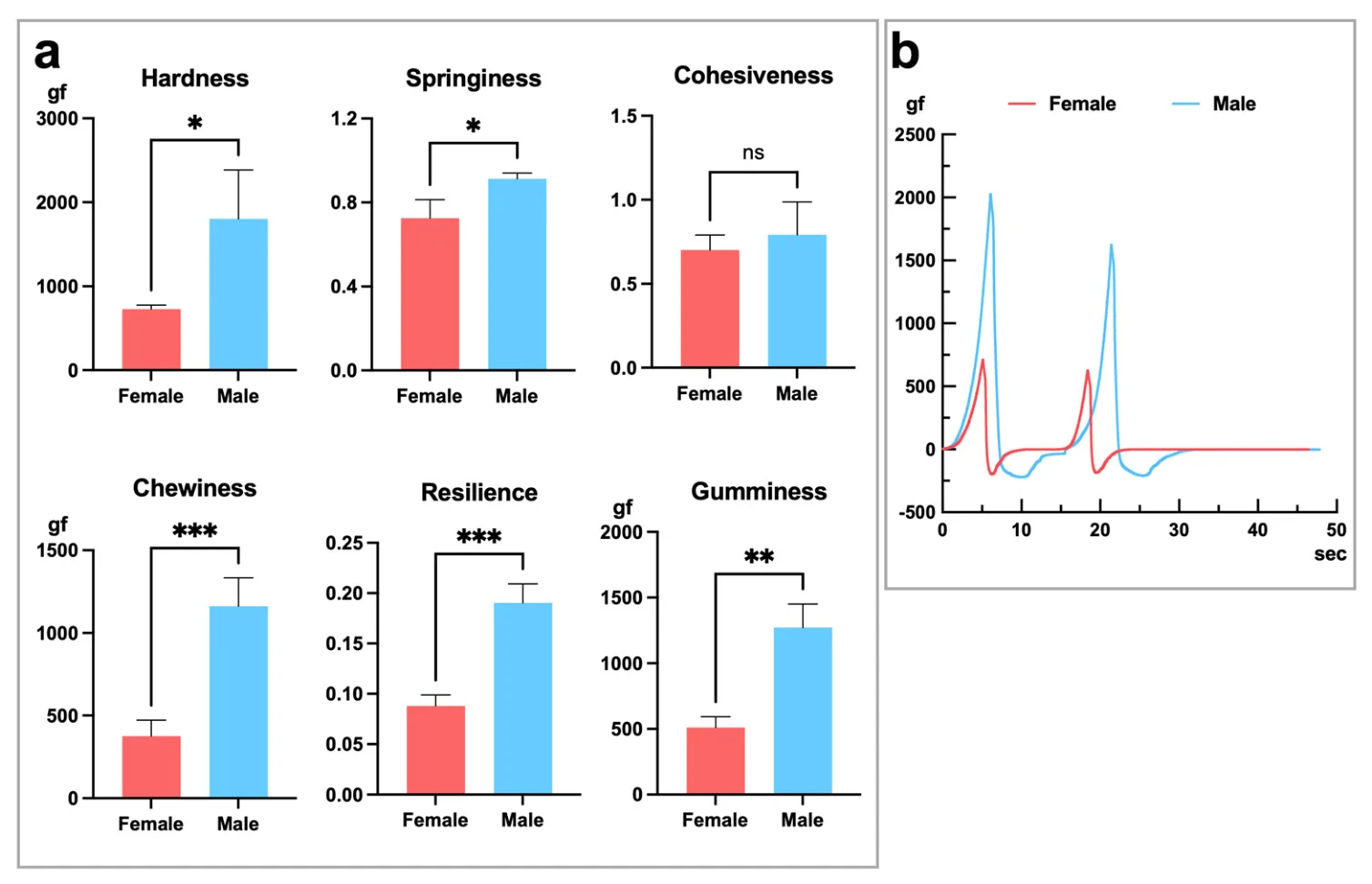
TPA of swim bladders from female and male Black-spotted Croaker (*Protonibea diacanthus*). (**a**) Comparison of texture parameters between sexes, including hardness (gf; gram-force), springiness (ratio), cohesiveness (ratio), chewiness (gf), resilience (ratio), and gumminess (gf). Data are presented as mean ± SD (*n* = 4 per sex). Asterisks indicate significant differences: * *p* < 0.05, ** *p* < 0.01, *** *p* < 0.001; “ns” denotes no significant difference (*p* > 0.05). (**b**) Representative force-time curves obtained from female and male swim bladder samples.

TPA was performed to compare the mechanical properties of male and female swim bladders (Figure 2a, Table S2). The measured parameters included hardness (gf), springiness (ratio), cohesiveness (ratio), chewiness (gf), resilience (ratio), and gumminess (gf). Male swim bladders exhibited significantly higher hardness (mean ± SD: 1804.33 ± 581.05 gf) than female ones (728.53 ± 46.81 gf). Springiness was also significantly greater in males (0.91 ± 0.03) than in females (0.73 ± 0.09). Cohesiveness did not differ between sexes (males: 0.79 ± 0.20; females: 0.70 ± 0.09). Chewiness was markedly higher in males (1161.18 ± 171.86 gf) than in females (374.68 ± 97.72 gf). Resilience also showed a significant male-biased difference (males: 0.19 ± 0.02; females: 0.09 ± 0.01). Gumminess was also higher in males (1272.14 ± 178.5 gf) than in females (511.41 ± 80.95 gf). These results indicate that the swim bladder of male *P. diacanthus* possesses superior textural properties, particularly in hardness, springiness, chewiness, resilience, and gumminess, all of which are critical attributes for fish maw quality grading and consumer preference. Representative force-time curves of female and male swim bladders are shown in Figure 2b.

### 3.3. Swim Bladder Nutrient Composition

#### 3.3.1. Proximate Composition and Collagen Content

**Figure 3:**
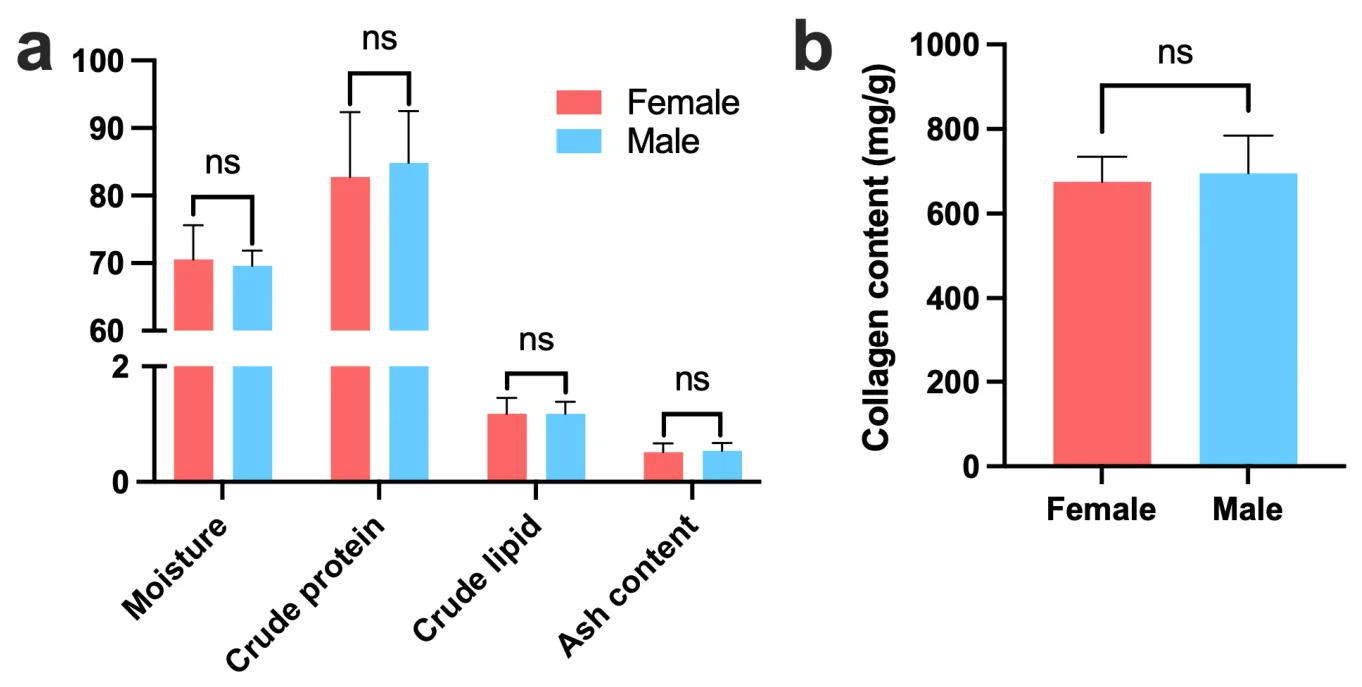
Proximate composition and collagen content of swim bladder from male and female Black-spotted Croaker (*Protonibea diacanthus*). (**a**) Moisture (expressed as % wet weight), crude protein, crude lipid, and ash (all expressed as % dry weight) of swim bladder. (**b**) Collagen content (mg/g dry weight) of swim bladder. Data are presented as mean ± SD (*n* = 4 per sex). “ns” denotes no significant difference (*p* > 0.05).

The proximate compositions and collagen content of swim bladders from both sexes are presented in Figure 3 and Table S3. Swim bladders of *P. diacanthus* were characterized by high protein and low lipid content. No significant differences were detected between females and males for moisture, crude protein, crude lipid, or ash content. Similarly, collagen content was high in both sexes (675.35 ± 58.82 mg/g dry weight in females vs. 695.43 ± 88.76 mg/g dry weight in males) and did not differ significantly between sexes (*p* > 0.05).

#### 3.3.2. Amino Acid Composition and Collagen Content

A total of 17 amino acids were identified and quantified in the swim bladders of both sexes (Table 1). Glycine was the most abundant amino acid, followed by proline and glutamic acid, consistent with the collagen-rich nature of swim bladder tissue. Among the amino acids, glutamic acid and leucine were significantly higher in males than in females (*p* < 0.05). Specifically, the glutamic acid content in male swim bladders was 13.11 ± 0.17%, compared to 12.58 ± 0.21% in females. Leucine content was 3.04 ± 0.10% in males and 2.71 ± 0.16% in females. No other amino acids showed significant sex-related differences.

#### 3.3.3. Fatty Acid Composition

The fatty acid profiles of swim bladders from both sexes are summarized in Table 2. Saturated fatty acids (SFAs) were the predominant class in both sexes, with palmitic acid (C16:0) and stearic acid (C18:0) being the most abundant individual fatty acids. Monounsaturated fatty acids (MUFAs) accounted for 8.69 ± 1.28% in males and 12.49 ± 1.92% in females, while polyunsaturated fatty acids (PUFAs) represented 12.10 ± 4.07% and 10.67 ± 4.39%, respectively. Notable PUFA components included eicosapentaenoic acid (EPA, C20:5n3; male: 3.05 ± 1.68%, female: 3.95 ± 1.73%) and docosahexaenoic acid (DHA, C22:6n3; male: 5.14 ± 1.85%, female: 3.87 ± 1.81%). Sex-specific differences were observed for several individual fatty acids. Female swim bladders contained significantly higher levels of C14:0, C16:1, and C18:3n3 than male swim bladders. Conversely, male swim bladders had significantly higher contents of C10:0, C14:1, C18:0, and C22:1n9.

#### 3.3.4. Mineral Element Composition

The mineral element profiles of male and female swim bladders are shown in Table 3. Swim bladders from both sexes contained substantial amounts of calcium, iron, zinc, and selenium. No significant differences in any individual mineral element were detected between sexes (all *p* > 0.05). However, calcium (Ca) showed a trend toward higher concentrations in males (79.53 ± 8.51 mg/kg) than in females (62.51 ± 8.49 mg/kg), although the difference did not reach statistical significance (*p* = 0.068). Trace heavy metal elements, including cadmium (Cd) and lead (Pb), were below national food safety standard limits in all samples, and mercury (Hg) was not detected.

### 3.4. Muscle Nutrient Composition

Table 4 presents the proximate composition, key amino acid indices, and main fatty acid classes of dorsal muscle from male and female *P. diacanthus*. Moisture, crude protein, crude lipid, and ash contents of dorsal muscle did not differ significantly between sexes (*p* > 0.05; Table 4). Males tended to have numerically higher crude protein (69.83 ± 6.77% vs. 65.10 ± 7.13% dry weight) and lower crude lipid (12.90 ± 6.15% vs. 20.38 ± 9.69% dry weight) than females, but these differences were not statistically significant.

Total amino acid (TAA) content and the levels of all individual amino acids were comparable between sexes (Table S4). The essential amino acid to total amino acid ratio (EAA/TAA) was 49.16 ± 0.31% in females and 48.60 ± 0.91% in males, with corresponding EAA/NEAA ratios of 96.68 ± 1.19% and 94.62 ± 3.44%; neither index differed significantly between sexes. Glutamic acid was the most abundant amino acid, followed by aspartic acid and lysine. When expressed relative to crude protein, all essential amino acids in the muscle of both sexes exceeded the adult human requirements recommended by the FAO/WHO/UNU (1985) reference pattern [36], indicating that *P. diacanthus* muscle provides high-quality protein for human nutrition.

No significant sex-related differences were detected in fatty acid classes or individual fatty acids (Table S5). Saturated fatty acids (SFA) accounted for 36–37% of total fatty acids, monounsaturated fatty acids (MUFA) for 19–20%, and polyunsaturated fatty acids (PUFA) for 43–45%. Docosahexaenoic acid (DHA, C22:6n-3) was the single most abundant fatty acid (26.65 ± 1.66% in females vs. 30.16 ± 5.56% in males), followed by palmitic acid (C16:0) and eicosapentaenoic acid (EPA, C20:5n-3). The PUFA/SFA ratio exceeded 1.1 in both sexes, which is considered favorable from a human cardiovascular health perspective. Although not statistically significant, males exhibited a numerically higher DHA/EPA ratio, suggesting a tendency toward sex-specific fatty acid partitioning that may warrant further investigation.

To better interpret these non-significant results, standardized effect sizes (Cohen’s d) were examined. Effect sizes for key composite quality indices, crude protein (d = 0.68) and crude lipid (d = 0.92), were medium-to-large, whereas most individual amino acid and fatty acid differences were small. Given the limited sample size (*n* = 4 per sex), larger studies are warranted to confirm whether these differences are robust.

## 4. Discussion

This study revealed pronounced sexual dimorphism in swim bladder texture of commercial-sized *P. diacanthus*, with males exhibiting markedly higher hardness, springiness, chewiness, gumminess, and resilience than females. In contrast, body weight, swim bladder weight, gross collagen content, and dorsal muscle nutrient composition did not differ significantly between the sexes. Subtle sex-specific differences were observed for a few amino acids (glutamic acid and leucine) and individual fatty acids of swim bladder. These results indicate that sex critically influences swim bladder quality but not muscle quality, providing important insights for sex-specific product utilization and selective breeding. At harvest, body weight and body length did not differ significantly between sexes, although females tended to be heavier and longer on average. This indicates that mixed-sex grow-out is currently adequate for biomass production in *P. diacanthus*. However, given the consistent female-biased growth dimorphism reported in several other farmed sciaenids, such as *N. albiflora* and *L. crocea* [19,22], controlled grow-out trials are warranted to determine whether a female growth advantage exists in this species. Swim bladder weight and the relative swim bladder weight (RSW) were also comparable between the sexes. These RSW values fall within the range reported for other sciaenids, such as the totoaba (*Totoaba macdonaldi*), whose swim bladder accounts for 1.31% to 1.39% of body wet weight under culture conditions [37]. The strong positive correlation between swim bladder weight and body weight observed in both sexes suggests that swim bladder size scales predictably with somatic growth regardless of sex.

The observed sexual dimorphism in swim bladder texture, where males exhibited significantly higher hardness, springiness, chewiness, resilience, and gumminess, aligns with recent findings in other sciaenid species. In *N. coibor*, a closely related species, male swim bladders also demonstrated superior toughness and textural properties compared to females [10,11]. This consistency across species suggests a potentially conserved biological mechanism within the Sciaenidae family that drives sex-specific differences in swim bladder quality. From a functional perspective, the swim bladder of sciaenids also acts as the main sound-producing organ, generating species-specific advertisement calls by rapidly contracting sonic muscles against the swim bladder wall during spawning [38]. The greater springiness and resilience of male swim bladders observed here may therefore contribute to more efficient sound production. The molecular basis of this sexual dimorphism was recently investigated by Zhang et al. confirmed these findings and further revealed through proteomic analysis that the highly expressed collagen XII in male swim bladders is likely a primary molecular factor contributing to this sexual dimorphism [11]. Our study extends these findings to *P. diacanthus*, a high-value commercial species, and confirms that the market preference for male fish maw [2,8] is based on measurable differences in texture.

Notably, the superior texture of male swim bladders was not accompanied by higher gross collagen content. Both sexes had comparably high collagen levels, consistent with the well-established collagen-rich nature of sciaenid swim bladders [11,39]. This finding suggests that the textural differences between the sexes are driven not by total collagen quantity, but rather by qualitative aspects of collagen structure, such as fiber organization, cross-linking density, and the composition of minor collagen types. Indeed, Zhang et al. showed that collagen fiber morphology differs markedly between male and female *N. coibor* swim bladders, with males exhibiting more uniformly shaped and densely packed collagen fibers [11]. Collagen XII, a fibril-associated collagen with interrupted triple helices, can bind to the surface of collagen fibrils via its collagenous domains and influence fibril spacing and organization [40]. The higher expression of genes of type XII (*col12a1*) collagen in male swim bladders may thus contribute to the greater hardness, chewiness, and overall toughness observed. Although proteomic analyses were beyond the scope of the present study, our findings are consistent with this mechanistic model and provide a strong rationale for future molecular investigations in *P. diacanthus*.

The swim bladders of both sexes were characterized by high protein and very low lipid content, in agreement with previous reports on *P. diacanthus* and other sciaenid swim bladders [11,41]. Glycine, proline, and glutamic acid were the three most abundant amino acids, a profile typical of type I collagen, which is the dominant protein in fish swim bladder tissue [42,43]. Functionally, glycine occupies every third position in the collagen triple helix, while proline (and its hydroxylated form, hydroxyproline) stabilizes the helical structure through pyrrolidine ring constraints [31,44]. Among the 17 detected amino acids, only glutamic acid and leucine were significantly elevated in males. Glutamic acid is a major component of collagen and contributes to the umami taste of fish maw products [45], and leucine is an essential branched-chain amino acid important for protein metabolism [46], suggesting that male swim bladders may offer modest sensory and nutritional advantages. Nevertheless, as expected for a collagen-dominated tissue, the EAA/TAA ratio of the swim bladder remained low, and the EAA/NEAA ratio was correspondingly low, reflecting the fact that over 40% of total amino acids were accounted for by glycine, proline, and hydroxyproline [44]. These indices are consistent with the nutritional profile of fish maw as a collagen-based functional food rather than a conventional dietary protein source. Sex-specific differences were observed for several individual fatty acids, possibly reflecting divergent lipid metabolism [47]. The notable presence of EPA and DHA in both sexes aligns with fatty acid profiles reported for other marine sciaenid swim bladders [37] and contributes to the nutritional value of *P. diacanthus* fish maw. Heavy metal concentrations (Cd, Pb, Hg) were well below Chinese national food safety limits in all samples, confirming the safety of swim bladder products from both sexes for consumption.

In contrast to the swim bladder, no statistically significant sex-related differences were detected in dorsal muscle proximate composition, amino acid profiles, or fatty acid profiles. However, effect size analysis revealed medium-to-large effects for key composite quality indices such as crude protein and crude lipid, suggesting that subtle sex-related differences in muscle composition may exist but were not detectable with the present sample size. The muscle of both sexes was of high nutritional quality, with an EAA/TAA ratio approaching 49%, all essential amino acids exceeding the FAO/WHO/UNU reference pattern [36], and a favorable fatty acid profile characterized by abundant DHA and a PUFA/SFA ratio above 1.1. These values are broadly consistent with those reported for other farmed sciaenids [48,49], indicating that *P. diacanthus* muscle is comparable to that of related high-value croakers. These nutritional indices were essentially identical between males and females, and stand in marked contrast to the low EAA/TAA and collagen-dominated amino acid profile of the swim bladder, underscoring the tissue-specific nature of sexual dimorphism in this species. The absence of sex-related differences in muscle nutritional quality in *P. diacanthus* indicates that fillet products from both males and females are nutritionally equivalent, simplifying processing and marketing strategies. From an aquaculture perspective, this means that sex-based sorting is unnecessary when the primary product target is muscle nutritional quality, and efforts can focus on maximizing overall biomass production. In contrast, when the high-value swim bladder is the primary product, an all-male culture strategy could yield a harvest of greater economic value. This highlights the potential benefits of developing monosex male production technologies for this species.

## 5. Conclusions

In conclusion, this study demonstrates that commercially sized *P. diacanthus* exhibit pronounced sexual dimorphism in swim bladder textural properties, with males showing substantially higher hardness, springiness, chewiness, gumminess, and resilience. But not in body weight, swim bladder size, gross collagen content, or muscle nutrient composition. These findings provide a scientific basis for sex-based grading and differential utilization of swim bladders in the fish maw industry, while confirming that muscle quality is equivalent between the sexes. The results contribute to a growing body of evidence that male-biased swim bladder texture is a common feature across commercially important sciaenid species and lay the groundwork for future research into the molecular mechanisms and potential dietary or genetic interventions to modulate swim bladder quality in aquaculture of this species.

## Figures and Tables

**Table 1:** Amino acid composition of swim bladder from male and female Black-spotted Croaker (*Protonibea diacanthus*).

Amino Acid	Female	Male
EAA		
Arg	8.43 ± 0.11	8.66 ± 0.13
Thr	2.78 ± 0.09	2.91 ± 0.06
Leu	2.71 ± 0.16	3.04 ± 0.10 *
Val	2.41 ± 0.18	2.69 ± 0.03
Lys	3.61 ± 0.05	3.68 ± 0.07
Phe	2.24 ± 0.03	2.21 ± 0.16
Ile	1.14 ± 0.11	1.31 ± 0.06
Met	1.34 ± 0.14	1.52 ± 0.10
His	0.76 ± 0.04	0.83 ± 0.02
NEAA		
**Gly**	20.53 ± 0.32	20.61 ± 0.46
**Pro**	20.08 ± 0.20	20.34 ± 0.35
Ser	2.55 ± 0.05	2.62 ± 0.11
Glu	12.58 ± 0.21	13.11 ± 0.17 *
Ala	12.02 ± 0.19	12.15 ± 0.20
Asp	5.89 ± 0.18	6.21 ± 0.11
Tyr	0.75 ± 0.13	0.88 ± 0.03
Cys	0.07 ± 0.03	0.12 ± 0.02
EAA/TAA (%)	26.09 ± 0.35	25.27 ± 0.21
EAA/NEAA (%)	35.3 ± 0.64	33.81 ± 0.38

Values are expressed as % dry weight. Data are presented as mean ± SD, *n* = 4 per sex. Asterisks indicate significant differences between sexes (* *p* < 0.05). Collagen-specific amino acids (Pro and Gly) are highlighted in bold. For essential amino acid (EAA), tryptophan was not determined. TAAs, total amino acids; EAAs, essential amino acids; NEAAs, non-essential amino acids.

**Table 2:** Fatty acid composition of swim bladder from male and female Black-spotted Croaker (*Protonibea diacanthus*).

Fatty Acid	Female	Male
SFA		
C6:0	0.05 ± 0.05	0.05 ± 0.02
C10:0	0.06 ± 0.01	0.11 ± 0.01 *
C11:0	0.02 ± 0.01	0.03 ± 0.01
C12:0	0.53 ± 0.09	0.67 ± 0.11
C13:0	0.17 ± 0.04	0.11 ± 0.03
C14:0	17.97 ± 2.61	10.06 ± 3.00 *
C15:0	1.50 ± 0.26	1.15 ± 0.03
C16:0	39.58 ± 1.94	43.31 ± 2.69
C17:0	1.27 ± 0.31	0.95 ± 0.19
C18:0	14.64 ± 2.29	21.92 ± 3.25 *
C20:0	0.69 ± 0.20	0.49 ± 0.06
C21:0	0.06 ± 0.03	0.08 ± 0.02
C22:0	0.23 ± 0.06	0.21 ± 0.04
C23:0	0.02 ± 0.01	0.01 ± 0.01
MUFA		
C14:1	0.10 ± 0.01	0.22 ± 0.08 *
C16:1	6.27 ± 1.05	3.33 ± 1.16 *
C18:1n9c	5.24 ± 1.17	4.41 ± 0.30
C20:1	0.46 ± 0.18	0.20 ± 0.06
C22:1n9	0.21 ± 0.03	0.39 ± 0.06 *
C24:1	0.21 ± 0.06	0.14 ± 0.00
PUFA		
C18:2n6c	0.72 ± 0.14	0.80 ± 0.07
C18:3n6	0.09 ± 0.04	0.06 ± 0.03
C18:3n3	0.33 ± 0.11	0.19 ± 0.05 *
C20:2	0.04 ± 0.00	0.03 ± 0.01
C20:3n6	0.05 ± 0.01	0.04 ± 0.00
C20:4n6	1.61 ± 0.71	2.79 ± 0.83
C20:3n3	0.02 ± 0.01	N.D.
C20:5n3 (EPA)	3.95 ± 1.73	3.05 ± 1.68
C22:6n3 (DHA)	3.87 ± 1.81	5.14 ± 1.85
SFA	76.80 ± 2.50	79.17 ± 3.93
MUFA	12.49 ± 1.92	8.69 ± 1.28
PUFA	10.67 ± 4.39	12.10 ± 4.07
PUFA/SFA	0.14 ± 0.06	0.15 ± 0.06

Values are expressed as % of total fatty acids. Data are presented as mean ± SD, *n* = 4 per sex. Asterisks indicate significant differences between sexes (* *p* < 0.05). The following fatty acids were not detected in either sex: C4:0, C8:0, C15:1, C17:1, C18:1n9t, C18:2n6t, C22:2 and C24:0. SFA, saturated fatty acid; MUFA, monounsaturated fatty acid; PUFA, polyunsaturated fatty acid; EPA, eicosapentaenoic acid; DHA, docosahexaenoic acid. N.D., not detected.

**Table 3:** Mineral element composition of swim bladder from male and female Black-spotted Croaker (*Protonibea diacanthus*).

Mineral Element	Female	Male
Mg	245.29 ± 52.81	225.61 ± 66.1
Ca	62.51 ± 8.49	79.53 ± 8.51
Mn	1.57 ± 0.36	2.04 ± 0.62
Fe	142.69 ± 54.67	132.88 ± 25.97
Cu	1.11 ± 0.34	0.88 ± 0.16
Zn	12.68 ± 3.31	12.09 ± 4.38
Se	0.55 ± 0.18	0.41 ± 0.22
Cd	0.003 ± 0.001	0.003 ± 0.002
Pb	0.07 ± 0.03	0.06 ± 0.01
Hg	N.D.	N.D.

Values are expressed as mg/kg dry weight. Data are presented as mean ± SD, *n* = 4 per sex. N.D. indicates not detected (below the limit of detection).

**Table 4:** Proximate composition, key amino acid indices, and main fatty acid classes of dorsal muscle from male and female Black-spotted Croaker (*Protonibea diacanthus*).

Parameter	Female	Male
Proximate composition		
Moisture (%)	61.27 ± 1.84	61.23 ± 3.57
Crude protein (% dry weight)	65.1 ± 7.13	69.83 ± 6.77
Crude lipid (% dry weight)	20.38 ± 9.69	12.9 ± 6.15
Ash (% dry weight)	3.93 ± 0.45	4.2 ± 0.38
Amino acid indices		
TAA (% dry weight)	61.51 ± 7.01	65.26 ± 6.35
EAA/TAA (%)	49.16 ± 0.31	48.6 ± 0.91
EAA/NEAA (%)	96.68 ± 1.19	94.62 ± 3.44
Fatty acid classes (%)		
SFA	37.29 ± 0.95	36.18 ± 2.8
MUFA	19.88 ± 0.51	18.88 ± 1.32
PUFA	42.83 ± 1.31	44.94 ± 3.99
EPA (C20:5n-3)	10.04 ± 1.1	9.04 ± 1.73
DHA (C22:6n-3)	26.65 ± 1.66	30.16 ± 5.56
PUFA/SFA	1.15 ± 0.06	1.25 ± 0.22

Data are presented as mean ± SD, *n* = 4 per sex. No significant differences were detected between sexes for any parameter (*p* > 0.05). TAA, total amino acids; EAA, essential amino acids; NEAA, non-essential amino acids; SFA, saturated fatty acids; MUFA, monounsaturated fatty acids; PUFA, polyunsaturated fatty acids; EPA, eicosapentaenoic acid; DHA, docosahexaenoic acid. Detailed individual amino acid and complete fatty acid profiles are provided in Supplementary Tables S4 and S5.

## Data Availability

The data that support the findings of this study are available from the corresponding authors upon reasonable request.

## Supplementary Materials

The following supporting information can be downloaded at: https://www.mdpi.com/article/10.3390/ani16162534/s1, Table S1: Body size and swim bladder dimensions of female and male Black-spotted Croaker (*Protonibea diacanthus*); Table S2: TPA of swim bladders from female and male Black-spotted Croaker (*Protonibea diacanthus*); Table S3: Proximate composition and collagen content of swim bladder from male and female Black-spotted Croaker (*Protonibea diacanthus*); Table S4: Amino acid composition of dorsal muscle from male and female Black-spotted Croaker (*Protonibea diacanthus*); Table S5: Fatty acid composition of dorsal muscle from male and female Black-spotted Croaker (*Protonibea diacanthus*).

## Abbreviations

The following abbreviations are used in this manuscript:

BW	Body weight
BL	Body length
SW	Swim bladder weight
RSW	Relative swim bladder weight
TPA	Texture profile analysis
SFA	Saturated fatty acid
MUFA	Monounsaturated fatty acid
PUFA	Polyunsaturated fatty acid
EPA	Eicosapentaenoic acid
DHA	Docosahexaenoic acid
