# Supplementary material for: Sexual Dimorphism in Swim Bladder Texture, Composition, and Muscle Nutrient Profile of Commercial-Sized Black-Spotted Croaker (Protonibea diacanthus)
Source: Animals (Basel). 2026 Aug 14;16(16):2534. doi: 10.3390/ani16162534 (PMC13508982; doi:10.3390/ani16162534)
Supplement: Supplementary file 1 [file animals-16-02534-s001.zip › animals-4492968-supplementary.pdf]

**Supplementary materials**

**Table S1. Body size and swim bladder dimensions of female and male Black-spotted Croaker (*Protonibea diacanthus*).**

| | Female | Male |
|---|---|---|
| BW (kg) | 13.5 ± 3.1 | 12.5 ± 2.3 |
| BL (cm) | 71.8 ± 5.3 | 70.1 ± 4.6 |
| SW (g) | 130.7 ± 34.4 | 122.9 ± 28.1 |
| RSW (%) | 0.97 ± 0.14 | 0.98 ± 0.10 |

Data are presented as mean ± SD, $n = 30$ per sex. No significant differences were detected between sexes for any parameter ($p > 0.05$).

**Table S2. TPA of swim bladders from female and male Black-spotted Croaker (*Protonibea diacanthus*).**

| | Female | Male | Intra-Sample CV (%) |
|---|---|---|---|
| Hardness (gf) | 728.53 ± 46.81 | 1804.33 ± 581.05 * | 13.5 ± 2.8 |
| Springiness | 0.73 ± 0.09 | 0.91 ± 0.03* | 3.9 ± 0.9 |
| Cohesiveness | 0.70 ± 0.09 | 0.79 ± 0.20 | 10.0 ± 4.3 |
| Chewiness (gf) | 374.68 ± 97.72 | 1161.18 ± 171.86*** | 15.9 ± 7.4 |
| Resilience | 0.09 ± 0.01 | 0.19 ± 0.02*** | 7.8 ± 0.3 |
| Gumminess (gf) | 511.41 ± 80.95 | 1272.14 ± 178.5** | 12.5 ± 3.7 |

Data are presented as mean ± SD, $n = 4$ per sex. Asterisks indicate significant differences between sexes (*$p < 0.05$, **$p < 0.01$, ***$p < 0.001$). Intra-Sample CV% is the mean coefficient of variation of three technical replicate measurements on each swim bladder sample.

**Table S3. Proximate composition and collagen content of swim bladder from male and female Black-spotted Croaker (*Protonibea diacanthus*).**

| | Female | Male |
|---|---|---|
| Moisture (%) | 70.55 ± 5.09 | 69.63 ± 2.25 |
| Crude protein (% dry weight) | 82.75 ± 9.63 | 84.83 ± 7.69 |
| Crude lipid (% dry weight) | 1.18 ± 0.27 | 1.18 ± 0.2 |
| Ash content (% dry weight) | 0.51 ± 0.15 | 0.54 ± 0.14 |
| Collagen (mg/g dry weight) | 675.35 ± 58.82 | 695.43 ± 88.76 |

Data are presented as mean ± SD, $n = 4$ per sex. No significant differences were detected between sexes for any parameter ($p > 0.05$).

**Table S4. Amino acid composition of dorsal muscle from male and female Black-spotted Croaker (*Protonibea diacanthus*).**

| Amino Acid | Female | Male |
|---|---|---|
| EAA | | |
| Lys | 5.85 ± 0.67 | 6.34 ± 0.65 |
| Leu | 4.85 ± 0.53 | 5.23 ± 0.47 |
| Arg | 3.94 ± 0.45 | 4.13 ± 0.4 |
| Val | 3.17 ± 0.32 | 3.39 ± 0.28 |
| Thr | 2.94 ± 0.33 | 3.14 ± 0.29 |
| Phe | 2.49 ± 0.3 | 2.69 ± 0.26 |
| Met | 2.2 ± 0.26 | 2.35 ± 0.22 |
| His | 1.61 ± 0.15 | 1.73 ± 0.14 |
| NEAA | | |
| Glu | 9.78 ± 1.12 | 10.48 ± 1 |
| Asp | 6.47 ± 0.76 | 6.93 ± 0.66 |
| Ala | 4.11 ± 0.54 | 4.26 ± 0.46 |
| Gly | 3.46 ± 0.71 | 3.31 ± 0.48 |
| Ile | 3.03 ± 0.32 | 3.26 ± 0.29 |
| Ser | 2.55 ± 0.3 | 2.72 ± 0.25 |
| Pro | 2.32 ± 0.4 | 2.27 ± 0.29 |
| Tyr | 2.06 ± 0.16 | 2.28 ± 0.19 |
| Cys | 0.69 ± 0.08 | 0.75 ± 0.06 |
| EAA/TAA (%) | 49.16 ± 0.31 | 48.6 ± 0.91 |
| EAA/NEAA (%) | 96.68 ± 1.19 | 94.62 ± 3.44 |

Values are expressed as % dry weight. Data are presented as mean ± SD, $n = 4$ per sex. No significant differences were detected between sexes for any parameter ($p > 0.05$). TAA, total amino acids; EAA, total essential amino acids; NEAA, total non-essential amino acids.

**Table S5. Fatty acid composition of dorsal muscle from male and female Black-spotted Croaker (*Protonibea diacanthus*).**

| Fatty Acid | Female | Male |
|---|---|---|
| SFA | | |
| C12:0 | 0.03 ± 0.01 | 0.03 ± 0.02 |
| C13:0 | 0.03 ± 0.01 | 0.03 ± 0.02 |
| C14:0 | 6.34 ± 0.71 | 5.32 ± 1.99 |
| C15:0 | 0.81 ± 0.08 | 0.69 ± 0.19 |
| C16:0 | 22.96 ± 0.89 | 22.03 ± 2.12 |
| C17:0 | 0.67 ± 0.04 | 0.64 ± 0.08 |
| C18:0 | 5.79 ± 0.61 | 6.86 ± 1.68 |
| C20:0 | 0.33 ± 0.02 | 0.31 ± 0.04 |
| C21:0 | N.D. | 0.01 ± 0.01 |
| C22:0 | 0.1 ± 0.02 | 0.12 ± 0.03 |
| C23:0 | 0.18 ± 0.02 | 0.11 ± 0.06 |
| MUFA | | |
| C14:1n5 | 0.04 ± 0.01 | 0.02 ± 0.03 |
| C16:1n7 | 5.15 ± 0.48 | 4.33 ± 1.23 |
| C17:1n7 | 0.23 ± 0.01 | 0.20 ± 0.01 |
| C18:1n9t | 0.15 ± 0.02 | 0.12 ± 0.03 |
| C18:1n9c | 10.75 ± 0.21 | 10.26 ± 0.5 |
| C20:1n9 | 1.71 ± 0.15 | 1.58 ± 0.29 |
| C22:1n9 | 0.34 ± 0.03 | 0.36 ± 0.01 |
| C24:1n9 | 1.52 ± 0.31 | 2.00 ± 0.67 |
| PUFA | | |
| C18:2n6c | 2.74 ± 0.66 | 2.32 ± 0.11 |
| C18:3n6 | 0.13 ± 0.02 | 0.12 ± 0.03 |
| C18:3n3 | 1.01 ± 0.05 | 0.86 ± 0.26 |
| C20:2n6 | 0.04 ± 0 | 0.03 ± 0.01 |
| C20:3n6 | 0.21 ± 0.01 | 0.23 ± 0.08 |
| C20:3n3 | 0.27 ± 0.02 | 0.22 ± 0.03 |
| C20:4n6 | 1.78 ± 0.13 | 1.99 ± 0.48 |
| C20:5n3 (EPA) | 10.04 ± 1.1 | 9.04 ± 1.73 |
| C22:6n3 (DHA) | 26.65 ± 1.66 | 30.16 ± 5.56 |
| SFA | 37.29 ± 0.95 | 36.18 ± 2.8 |
| MUFA | 19.88 ± 0.51 | 18.88 ± 1.32 |
| PUFA | 42.83 ± 1.31 | 44.94 ± 3.99 |
| PUFA/SFA | 1.15 ± 0.06 | 1.25 ± 0.22 |

Values are expressed as % of total fatty acids. Data are presented as mean ± SD, $n = 4$ per sex. N.D. indicates not detected (below the limit of detection). No significant differences were detected between sexes for any parameter ($p > 0.05$). SFA, saturated fatty acid; MUFA, monounsaturated fatty acid; PUFA, polyunsaturated fatty acid; EPA, eicosapentaenoic acid; DHA, docosahexaenoic acid. N.D., not detected.
